# Age-Related Behavioral Phenotype of an Astrocytic Monoamine Oxidase-B Transgenic Mouse Model of Parkinson’s Disease

**DOI:** 10.1371/journal.pone.0054200

**Published:** 2013-01-10

**Authors:** Christopher A. Lieu, Shankar J. Chinta, Anand Rane, Julie K. Andersen

**Affiliations:** 1 Buck Institute for Research on Aging, Novato, California, United States of America; The University of Melbourne, Australia

## Abstract

We have previously shown that increases in astrocytic monoamine oxidase-B (MAO-B) expression, mimicking that which occurs with aging and in neurodegenerative disease, in a doxycycline (dox)-inducible transgenic mouse model evokes neuropathological similarities to what is observed in the human parkinsonian brain. Additional behavioral and neuropathological studies could provide further validation for its usage as a model for Parkinson’s disease (PD). In the present study, we utilized a battery of behavioral tests to evaluate age-related phenotype in this model. In the open field test, we found that dox-induction impaired motor ability with decreases in movement and ambulatory function as well as diminished stereotypical, repetitive movement episodes in both young and old mice. Older mice also showed decreased motor performance in the pole test when compared to younger mice. Furthermore, dox-induced older mice displayed severe hindlimb clasping and the most significant loss of dopamine (DA) in the striatum when compared to young and non-induced animals. Additionally, increased MAO-B activity significantly correlated with decreased expression of striatal DA. The results of our study further confirms that the dox-inducible astrocytic MAO-B transgenic mouse displays similar age-related behavioral and neuropathological features to other models of PD, and could serve as a useful tool to study PD pathophysiology and for the evaluation of therapeutic interventions.

## Introduction

Parkinson’s disease (PD) is a common, neurodegenerative disorder characterized by bradykinesia (slowness of movement), muscle rigidity, postural instability and resting tremor. The main pathological feature of PD is chronic progressive loss of dopaminergic nigrostriatal neurons and loss of dopamine (DA) to the striatum. A significant loss of these neurons and subsequent striatal DA is necessary before symptoms occur resulting in a clinical diagnosis of the disorder.

Monoamine oxidase-B (MAO-B) is an enzyme found in astrocytes and has been implicated in the neurodegenerative process associated with aging and in neurodegenerative diseases including Parkinson’s and Alzheimer’s disease [1]. Age-related increases in MAO-B expression is associated with increases in free radical damage and reactive oxygen species (ROS) [2]–[5]. This increase in free radicals and ROS via age-related increases in MAO-B expression has been reported to lead to decreases in neuronal mitochondrial function, deterioration of substantia nigra dopaminergic neuron viability, and ultimately leads to cell death [6], [7] and motor impairment. In contrast, MAO-B inhibitors are shown to prevent dopaminergic neuron degeneration [8], [9] and decrease parkinsonian symptoms [10]. Additionally, MAO-B knock-out mice demonstrate increases in prolonged locomotor activity and increased stress-related mobility when compared to wild-type mice [11], [12]. Taken together, it is evident that MAO-B plays an important role in the progressive nature of neurodegenerative diseases and subsequent behavioral pathophysiology.

We have previously shown that inducible elevation and subsequent increases in activity of astrocytic MAO-B within transgenic mice by treatment with doxycycline (dox), mimicking that which occurs with age and neurodegenerative disease, results in brain pathology similar to that reported in the human PD midbrain [13], [14]. This includes loss of dopaminergic nigrostriatal neurons, impairments in mitochondrial function and increases in oxidative stress and ROS levels. However, it is currently unclear to what extent these animals display parkinsonian behavioral characteristics. Further behavioral evaluation of this transgenic mouse could add to its validity as a model for PD. Therefore, in the present study, we challenged this mouse model at young and old ages to a battery of behavioral tests which have been utilized in other neurodegenerative and PD mouse models to determine if it displays a similar phenotype to other parkinsonian models and to human PD.

## Materials and Methods

### Ethics Statement

All experiments were carried out according to the National Institute of Health Guide for the Care and Use of Laboratory Animals (NIH Publications No. 80–23, revised 1978). The protocol was approved by the Institutional Animal Care and Use Committee (IACUC) of the Buck Institute for Research on Aging (IACUC Protocol #: 10070). All efforts were made to decrease animal suffering, to reduce the number of animals in the study and to utilize alternatives to *in vivo* techniques.

### Animals

Dox-inducible astrocytic MAO-B transgenic mice generated in the C57BL/6 background and bred to homozygosity were utilized in this study [13], [14]. Animals were kept on a 12 hr light/dark cycle, and had free access to food and water. Mice were aged and separated into four different groups: (1) 6 month old with dox treatment, (2) 6 month old without dox treatment, (3) 14 month old with dox treatment, and (4) 14 month old without dox treatment (n = 4–6 per group). To induce selective elevation of MAO-B levels within astrocytes, dox was given at 0.5 g/kg/day in pre-mixed Purina chow (Research Diets) for 12 weeks. Non-dox treated control transgenic animals were fed normal chow.

### Behavioral Tests

#### Open field spontaneous activity

To monitor spontaneous activity, mice were placed in an open field chamber and allowed to explore freely over a 10 minute period using a Tru Scan photobeam apparatus (Colbourn Instruments) as previously described by our laboratory [13]. Using the Tru Scan software, we evaluated floor plane movement, ambulation, resting and repetitive stereotypy (e.g. grooming, sniffing, and head-bobbing) as determined by the software parameters [15], [16].

#### Hindlimb clasping

Hindlimb clasping has been shown to occur in various neurodegenerative mouse models [17], [18]. For this test, mice were suspended by the base of the tail and videotaped for 10–15 seconds. Three separate trials were taken over three consecutive days. Hindlimb clasping was rated from 0 to 3 based on severity: 0 = hindlimbs splayed outward and away from the abdomen, 1 = one hindlimb retracted inwards towards the abdomen for at least 50% of the observation period, 2 = both hindlimbs partially retracted inwards towards the abdomen for at least 50% of the observation period, 3 = both hindlimbs completely retracted inwards towards the abdomen for at least 50% of the observation period. Scores of 0.5 were utilized when appropriate. Hindlimb clasping severity scores were added together for the three separate trials.

#### Pole test

The pole test has been utilized to measure motor coordination and balance in mouse models of PD [18]–[20]. In this test, animals were placed on top of a rough-surfaced wooden pole (50 cm in length and 1 cm in diameter) and allowed to descend to the base of the pole. Mice were initially habituated and trained the day prior to testing. On testing day, animals were placed head-up on the top of the pole. The time it took for the animal to turn its head downwards (movement initiation) and descend the entire length of the pole was taken. The best performance for each animal over five consecutive trials was subsequently recorded.

### Analysis of DA Levels and MAO-B Activity

Animals were euthanized after behavioral testing and brains were harvested then stored in −80°C for analysis of striatal DA levels and MAO-B activity. The striatum was isolated and sent to the Neurochemistry Core of the Center for Molecular Neuroscience at Vanderbilt University (Nashville, TN) for DA analysis. Cortical regions were isolated and analyzed for MAO-B activity utilizing the Amplex Red Monoamine Oxidase Assay Kit (Molecular Probes). Briefly, representative cortical regions from aged 14 month old animals were sonicated on ice with Tris-Cl then centrifuged at 4°C. Protein supernatant amount was then measured for equivalent amounts of protein (20 µg) to be used in the assay. The procedure was performed as per the manufacturer’s protocol using benzylamine as the substrate specific for MAO-B enzymatic activity. The reaction was incubated for 60 min at room temperature then analyzed on a fluorescence microplate reader using excitation at 560 nm and emission detection at 590 nm.

### Statistics

Two-way (age, 6 month versus 14 month; treatment, Dox versus No Dox), non-parametric analysis of variance using post-tests, and Student’s t-test was utilized in our studies when appropriate. Regression analysis was used to correlate MAO-B activity and DA striatal levels (Graphpad Prism). Significance was set at *p*<0.05. Data are expressed mean ± SEM.

## Results

### Open Field Spontaneous Movement Activity

**Figure 1:**
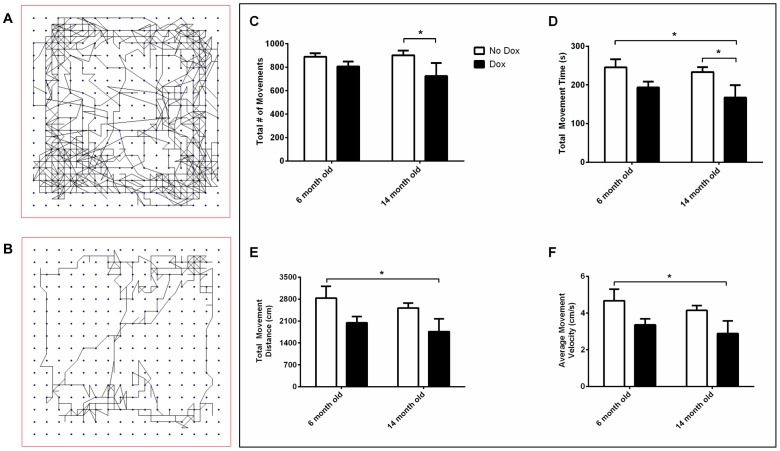
Open Field Movement Activity. Example of floor plane track plot for (A) non-induced and (B) induced astrocytic MAO-B transgenic mouse. Dox induction of astrocytic MAO-B transgenic mice decreases (C) total number of movements, (D) total movement time (s), (E) total movement distance (cm), and (F) average movement velocity (cm/s). *p<0.05.

Figure 1 shows the results for various spontaneous movement parameters during open field activity. Animals treated with dox to induce increased MAO-B expression showed less total numbers of overall movements, total movement time, total movement distance and average movement velocity when compared to non-induced animals (see Fig 1A and 1B for example of floor plane track plot). For total numbers of movement (Fig 1C), there was a significant main effect for treatment, *F*(1, 16) = 5.15, *p* = 0.04, but not a significant main effect for age, *F*(1, 16) = 0.36, n.s., or age X treatment interaction, *F*(1, 16) = 0.72, n.s. For total movement time (Fig 1D), there was a significant main effect for treatment, *F*(1, 16) = 8.84, *p* = 0.009, but not a significant main effect for age, *F*(1, 16) = 0.92, n.s., or age X treatment interaction, *F*(1, 16) = 0.13, n.s. For total movement distance (Fig 1E), there was a significant main effect for treatment, *F*(1, 16) = 7.23, *p* = 0.02, but not a significant main effect for age, *F*(1, 16) = 1.09, n.s., or age X treatment interaction, *F*(1, 16) = 0.00, n.s. For average movement velocity (Fig 1F), there was a significant main effect for treatment, *F*(1, 16) = 7.29, *p* = 0.02. A significant main effect for age, *F*(1, 16) = 1.10, n.s., and age X treatment interaction, *F*(1, 16) = 0.00, n.s. was not identified.

### Open Field Spontaneous Ambulatory Activity

**Figure 2:**
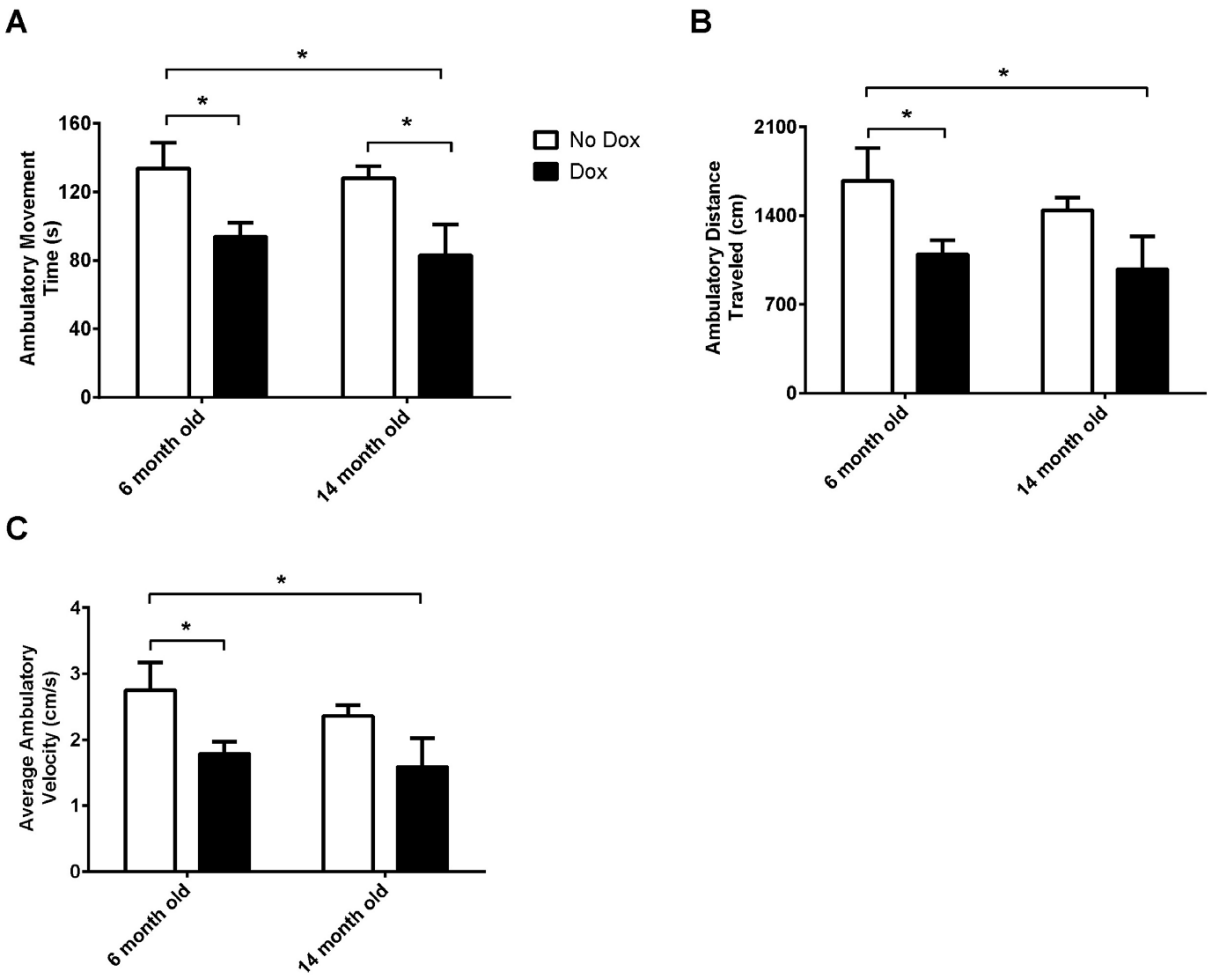
Open Field Ambulation Activity. Dox induction of astrocytic MAO-B transgenic mice decreases (A) ambulatory movement time (s), (B) ambulatory distance traveled (cm), and (C) average ambulatory velocity (cm/s). *p<0.05.

As shown in Figure 2, ambulation and related parameters in the open field were affected by dox induction. Here we show that ambulatory movement time, ambulatory distance traveled and average ambulatory velocity are all decreased in dox-induced animals when compared to non-induced animals. For average ambulatory movement time (Fig 2A), there was a significant main effect for treatment, *F*(1, 16) = 12.41, *p* = 0.003, but not a significant main effect for age, *F*(1, 16) = 0.47, n.s., or age X treatment interaction, *F*(1, 16) = 0.04, n.s. For ambulatory distance traveled (Fig 2B), there was a significant main effect for treatment, *F*(1, 16) = 7.92, *p* = 0.01, but not a significant main effect for age, *F*(1, 16) = 0.90, n.s., or age X treatment interaction, *F*(1, 16) = 0.09, n.s. For average ambulatory velocity (Fig 2C), there was a significant main effect for treatment, *F*(1, 16) = 7.94, *p* = 0.01. A significant main effect for age, *F*(1, 16) = 0.91, n.s., and age X treatment interaction, *F*(1, 16) = 0.10, n.s. was not seen.

### Open Field Resting and Stereotypy

**Figure 3:**
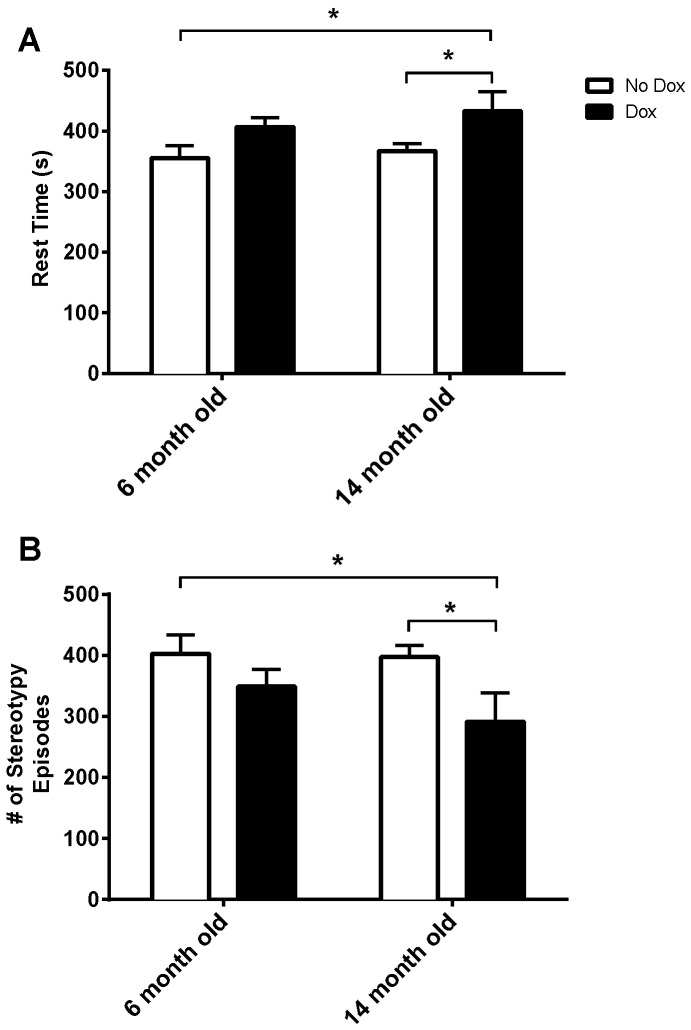
Rest Time and Stereotypy. Dox induction of astrocytic MAO-B transgenic mice increases (A) rest time (s) and decreases (B) the number of stereotypy episodes. *p<0.05.

In Figure 3A and 3B, we evaluated resting time and stereotypy episodes in our animals in the open field analysis. We found that dox induction increases resting time and decreases the number of stereotypy episodes, which have been described as including grooming, sniffing, and head-bobbing [15], [16]. For resting time (Fig 3A), there was a significant main effect for treatment, *F*(1, 16) = 8.84, *p* = 0.009. There was no significant main effect for age, *F*(1, 16) = 0.92, n.s., or age X treatment interaction, *F*(1, 16) = 0.13, n.s. For stereotypical movement episodes (Fig 3B), there was a significant main effect for treatment, *F*(1, 16) = 6.84, *p* = 0.02, but no significant main effect for age, *F*(1, 16) = 1.07, n.s., or age X treatment interaction, *F*(1, 16) = 0.73, n.s.

### Hindlimb Clasping and Pole Test Behavior

**Figure 4:**
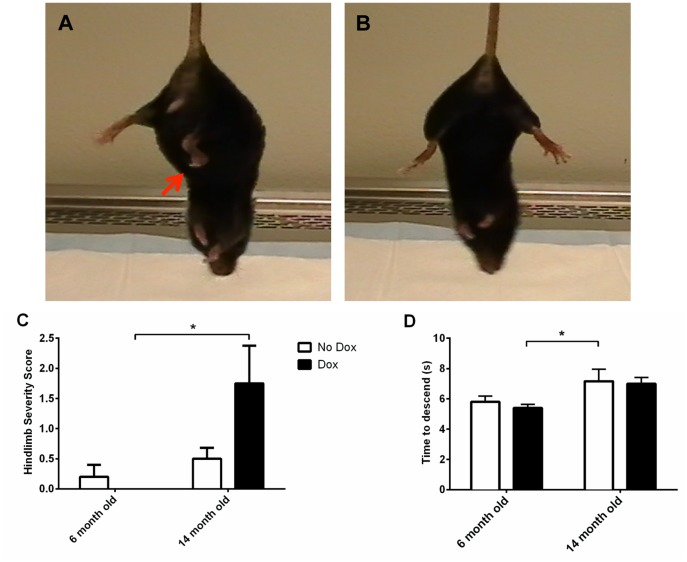
Hindlimb clasping and pole test. (A) Example of dox-induced hindlimb clasping behavior towards the abdomen (red arrow) and (B) lack of hindlimb clasping (hindlimbs splayed outwards away from abdomen) in non-induced animals. (C) Age-related dox induction causes increased hindlimb clasp severity. (D) Age-related decreases of motor coordination and balance in the pole test at 14 months. *p<0.05.

Hindlimb clasping was most severe in 14 month old dox-induced animals (see Fig 4A and 4B for example). As shown in Fig 4C, 14 month old dox-induced animals demonstrated severe hindlimb clasping behavior compared to the other groups (non-parametric ANOVA, *p* = 0.04). We found no changes in movement initiation as determined by turning ability in the pole test amongst the four different groups (data not shown). However, we did find a significant main effect for age, *F*(1, 16) = 6.94, *p* = 0.02, in the performance of the pole test when animals descended the length of the pole (Fig 4D). There was no main effect for treatment, *F*(1, 16) = 0.25, n.s., or age X treatment interaction, *F*(1, 16) = 0.04, n.s.

### Dopamine (DA) Levels in the Striatum and MAO-B Activity

**Figure 5:**
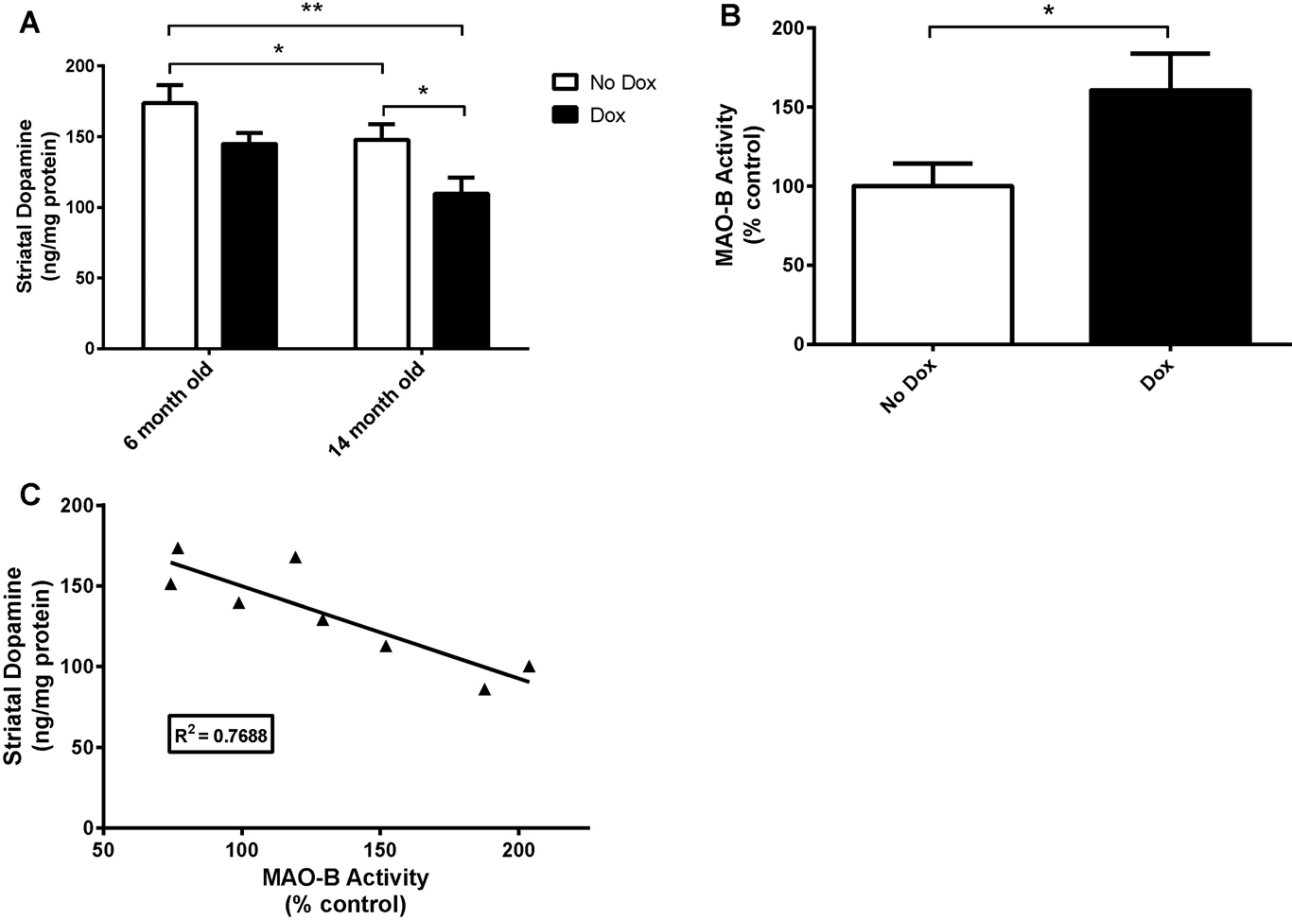
Striatal dopamine levels and confirmation of MAO-B activity. (A) Age and treatment-related decreases of DA levels in the striatum. (B) Confirmation of dox-related increases in MAO-B activity in aged animals (n = 4 per group; expressed as percent no-dox control). (C) Regression of MAO-B activity against striatal DA levels in aged animals (n = 8). *p<0.05 and **p<0.01.

DA levels in the striatum were significantly decreased in 14 month old dox-induced animals when compared to the other groups (Fig 5A). For DA levels, we found a significant main effect for age, *F*(1, 14) = 8.17, *p* = 0.01 and for treatment, *F*(1,14) = 9.80, *p* = 0.007. There was no main effect for age X treatment interaction, *F*(1, 14) = 0.19, n.s. To confirm MAO-B expression, we evaluated MAO-B enzymatic activity in representative aged animals (Fig 5B). We found that MAO-B activity significantly increased with dox treatment (*p* = 0.02). Furthermore, the correlation of MAO-B activity against striatal DA levels was significant (*p* = 0.004), with an R^2^ value of 0.7688 (Fig 5C).

## Discussion

Our initial studies in this model confirmed multiple neuropathological features similar to that in PD patients and other preclinical models of PD [13], [14]. MAO-B elevation caused a significant age-dependent loss of tyrosine hydroxylase (TH) dopaminergic neurons in the substantia nigra. Furthermore, older animals (14 months old) had an enhanced loss of TH+ neurons compared with younger mice (3–4 months old). Increased MAO-B expression also induced elevation in substantia nigra microglia activation. Additionally, anti-oxidant, anti-inflammatory, and MAO-B inhibitor treatment to such animals was found to be neuroprotective, indicating its validity as a model to test therapeutic interventions [13], [14].

In the present study, we established the parkinsonian behavioral properties of our astrocytic MAO-B transgenic mouse model using multiple behavioral parameters. Initial open field studies showed a 32–35% loss of ambulatory motor function in dox-induced MAO-B mice compared to non-induced MAO-B [13]. Similarly, we found treatment and age-dependent decreases in ambulatory function in addition to other behavioral variables (movement, resting and stereotypy) in the open field test. We also saw an age and treatment related increase in hindlimb clasping behavior, which is present in other neurodegenerative and PD mouse models [17], [18]. However, we did not see a dox-induced effect in the pole test, but rather identified an age-related effect. Furthermore, these animals did not show a deficit in movement initiation as measured by turning ability in the pole test. Others have identified movement initiation deficit using the forced stepping test in MPTP-treated mice and hemiparkinsonian 6-hydroxydopamine lesioned rats [21], [22]. This test is widely used to measure hemispheric differences in unilaterally lesioned rats. It is unclear if the forced stepping test is sensitive in our MAO-B model but, to our knowledge, this specific behavior is not widely utilized in other PD transgenic mouse models. Future studies that evaluate other motor initiation behaviors such as the adhesive removal test [20] which are validated in transgenic PD animals may prove to be sensitive in the MAO-B transgenic model. In addition to multiple behavioral deficits in the MAO-B transgenic mouse, we also showed that decreases in DA in the striatum was most severe in aged dox-treated mice, similar to previous reports of PD mouse models [13], [23], [24]. Taken together, these studies confirm that the astrocytic MAO-B transgenic model shows age-related neuropathological properties and multiple motor behavioral deficits similar to those exhibited in PD patients and preclinical models.

In conclusion, we demonstrate in the present study that increased expression of astrocytic MAO-B in transgenic mice causes many of the behavioral phenotypes found in other mouse models of PD. Along with our earlier studies in this model, it is clear that increases in astrocyte MAO-B expression is detrimental to neuronal function and can lead to multiple behavioral deficits. These results confirm that this mouse model can be useful to study PD-associated pathophysiology and testing of preclinical therapeutics.
